# Left atrial appendage volume as a prognostic Indicator of long-term mortality in Cancer survivors treated with thoracic radiation

**DOI:** 10.1186/s40959-023-00155-4

**Published:** 2023-01-14

**Authors:** Meer R. Zafar, Ashutosh Sharma, Sunitha Shyam Sunder, Badri Karthikeyan, Makoto Nagahama, Andrew Atia, Ronak Bahuva, Saraswati Pokharel, Vijay Iyer, Sharma Kattel, Umesh C. Sharma

**Affiliations:** 1 Department of Medicine, Division of Cardiology, Jacob’s School of Medicine and Biomedical Sciences, Buffalo, NY USA; 2 Department of Medicine at the Icahn School of Medicine, Mount Sinai Health System, New York, NY USA; 3 Division of Cardiology, Lehigh Valley Health Network, Allentown, PA USA; 4 Department of Pathology and Laboratory Medicine, Roswell Park Comprehensive Cancer Centre, Buffalo, NY USA; 5 Department of Medicine, Division of Cardiology, Yale School of Medicine, New Haven, CT USA; 6 Advanced Cardiovascular Imaging, Division of Cardiovascular Medicine, 875 Ellicott Street, Buffalo, New York, 14203 USA

**Keywords:** Cancer survivors, Cardiac CT, Left atrial appendage, Mortality, Radiotherapy

## Abstract

**Background:**

Cancer survivors with prior chest radiation therapy (CXRT) frequently present with atrial fibrillation, heart failure, and have higher overall long-term mortality. There are no data examining the utility of left atrial (LA) and LA appendage (LAA) volume-indices to predict clinical outcomes in these patients.

**Objectives:**

We examined the prognostic value of cardiac phase-dependent 3-D volume-rendered cardiac computerized tomography (CT)-derived LA and LAA volume-indices to predict mortality and major adverse cardiac events (MACE) in cancer survivors treated with thoracic irradiation.

**Method:**

We screened 625 consecutive patients with severe aortic stenosis who had undergone transcatheter aortic valve replacement from 2012 to 2017. Based on the gated cardiac CT image quality, we included 184 patients (CXRT:43, non-CXRT:141) for further analysis. We utilized multiplane-3D-reconstructed cardiac CT images to calculate LA and LAA volume-indices, and examined the prognostic role of CCT-derived LA and LAA volume-indices in predicting the all-cause mortality, cardiovascular (CV) mortality, and MACE. We used multivariate cox-proportional hazard analysis to identify the clinical predictors of survival.

**Results:**

Overall, the CXRT group had significantly elevated LAA volume-index compared to non-CXRT group (CXRT:11.2 ± 8.9 ml/m^2^; non-CXRT:8.6 ± 4.5 ml/m^2^, *p* = 0.03). On multivariate cox-proportional hazard analysis, the elevated LAA volume and LAA volume-index were the strongest predictors of reduced survival in CXRT group compared to non-CXRT group (LAA volume: RR = 1.03,95% CI 1.0–1.01, *p* = 0.01; and LAA volume index: RR = 1.05, 95% CI 1.0–1.01, p = 0.03). LAA volume > 21.9 ml was associated with increased mortality. In contrast, LA volume was not a significant predictor of mortality.

**Conclusion:**

We describe a novel technique to assess LA and LAA volume using 3-D volume-rendered cardiac CT. This study shows enlarged LAA volume rather than LA volume carries a poor prognosis in cancer-survivors treated with prior CXRT. Compared to conventionally reported markers, LAA volume of > 21.9 ml was incremental to that of other risk factors.

## Introduction

The cancer survival and recurrence data support the merit of ionizing radiation therapy in treating most common thoracic malignancies including breast and lung cancers [1]. Despite the safety advances achieved over the past decades with reduced radiation dose and improved shielding of normal tissues, there is increasing incidence of potentially fatal cardiovascular events in the breast cancer and Hodgkin’s lymphoma survivors [2–5]. Previous research using the Surveillance, Epidemiology, and End Results (SEER) database, showed higher incidence of late cardiac death in cancer survivors treated with radiotherapy [6]. Recent studies from our group also reported higher incidence of mortality and heart failure exacerbation in patients with prior CXRT compared to patients without CXRT [7, 8]. In this context, earlier detection of cardiac damage is crucial to better therapeutic adjudication of susceptible patients.

Previous studies of radiation-associated heart disease demonstrated that the incidence of heart damage varies with the dose, fractionation, and irradiated volume of the heart [5, 9]. In particular, anterior left-sided chambers are more susceptible to be exposed during the radiotherapy of the anterior thoracic structures, especially left-sided breast cancers [5, 9, 10]. The left atrial appendage (LAA) is a hook-like true diverticulum of the left atrium (LA). It has a heterogeneous composition with smooth-walled body and pectinate muscles with parallel orientation [11]. Prior studies also reported that LAA is more distensible than the LA and therefore, can retain larger blood volume when the LA pressure is elevated [12, 13]. However, the LAA continues to remain neglected and there are limited methods to quantify LAA volumes and limited data studying the risk predictive role of LAA volume or LAA volume indices.

Since the LA is the posterior-most cardiac chamber, there are discrepant views on whether LA is directly affected by cancer radiotherapy of the anterior structures [14–16]. Unanticipated LA irradiation is possible in patients undergoing radiotherapy for posterior mediastinal tumors, but the most common radiation algorithms developed for breast cancer therapy are limited to only anterior aspect of left atrium, which frequently affects LA appendage and anterior pulmonary venous ostia [2, 17] . The transthoracic echocardiogram, which is the most commonly used imaging modality to examine cardiac morphology and function cannot clearly visualize LAA. However, routine utilization of retrospectively-gated cardiac CT for pre-procedure analysis of cardiac morphology and function in patients undergoing valvular procedures, has enabled us to reliably examine LA, LA appendage and pulmonary venous morphology.

Because of the growing interest in the LAA remodeling and its role in the pathogenesis of major cardiovascular events (MACE), we sought to examine the association between the LAA volume indices in cancer survivors treated with thoracic irradiation. To this end, we have designed this study focusing on the volumetric assessment of LA, LAA and pulmonary veins calculated through a 320-slice gated CT scanner with a comprehensive 3-D volume rendered post-processing algorithms in subjects that survived various cancers after receiving thoracic radiotherapy. In particular, we analyzed the outcomes associated with LA and LAA volume-indices in radiation-exposed cohorts with a hypothesis that higher LA and LAA volume-indices portend higher mortality and less freedom from cardiovascular adverse events.

## Methods

We studied patients who had initially presented with aortic stenosis (AS) and had undergone transcatheter valve replacement (TAVR) from 2012 to 2017. All patients were selected from a single, tertiary care center, Buffalo General Hospital (BGH) and Gates Vascular Institute (GVI) in Buffalo, New York. The studies involving human participants were reviewed and approved by the University at Buffalo Institutional Review Board. Written informed consent for participation was not required for this study in accordance with the national legislation and the institutional requirements.

### Study population and design

We screened 625 patients who had undergone TAVR procedure at our institution. All patients underwent cardiac CT with a retrospective gating protocol preceding TAVR. Of the 625 patients, we were unable to perform 3-D volume rendering of the LA and LAA in 328 subjects (suboptimal images were rejected by the analytical software). The sub-optimal image quality was due to patient- or post-processing related factors. The other 113 patients were excluded due to atrial fibrillation. Therefore, we limited this study to 184 subjects meeting the following criteria: A) Study Population: Patients with history of thoracic malignancy (mostly lung, breast or esophageal) treated with chest radiotherapy and severe AS, B) Controls: Patients with severe AS without a history of prior radiation therapy. All patients underwent multimodality cardiac imaging including Electrocardiographic (ECG)-gated cardiac CT angiography with a retrospective gating protocol. Since the LA/LAA volumetric assessments were unpredictable with atrial fibrillation (AF), those patients are excluded from further analyses. Overall, the study subjects were divided into two groups. The first group (CXRT; *N* = 43) had prior history of radiation therapy for thoracic malignancy. The second control group (non-CXRT; *N* = 141) had no prior history of thoracic radiation therapy.

The dose and field of chest radiation varied depending upon the type and laterality of cancer being treated. For the breast cancer patients, patients that underwent either whole breast irradiation (either side, short course or hypo-fractionated whole breast, and patients that received post-mastectomy radiation therapy) were included. For other thoracic cancer types including Hodgkin’s lymphoma, the external beam radiation (including extended, mantle and inverted fields) within the thoracic cavity was considered as the chest irradiation. As per the NCCN guidelines, for the left sided breast cancers, up to 10% of the cardiac silhouette are exposed to 25Gy radiation. Similarly, the right sided breast, esophageal and lung cancers received up to 2, 30 and 25% radiation field exposure, respectively. As for the distribution in left sided breast cancer patients, anterior left portion of the heart, including left anterior descending (LAD) coronary artery, pericardium and anterior wall of the left ventricle showed increased risk of exposure. In lung cancers, the exposure varied depending upon the nodal involvement and the location of the tumors.

### Cardiac CT imaging protocols

Pre-procedural imaging with ECG gated cardiac CT to assess the prosthetic valve size, and coronary ostial height for procedural planning is a standard clinical practice for patients undergoing TAVR implant. A retrospectively gated cardiac CT allows accurate determination of aortic annular dimensions and perimeter area under different phases of the cardiac cycle. The imaging was performed with 320-multidetector scanner (Aquilion ONE, Toshiba Medical System, Japan) with iodinated contrast in the craniocaudal direction and Electrocardiography (ECG)-gated during breath hold. Bolus tracking technique was performed visually with region of interest in the left side of the heart. Image datasets were transferred to a remote workstation equipped with semi-automated post-processing software (Aquarius, TeraRecon, San Mateo, CA, USA) for further analysis and interpretation. Images were reconstructed at 0.5 mm with 0.4 mm increments. The cardiac CTs are interpreted by Society of Cardiovascular Computerized Tomography (SCCT) Board-Certified cardiologists.

### 3-D volumetric quantification of the LA and LAA volume changes per 10% increments of the cardiac cycle

The existing literature reports LA volume changes over the different phases of cardiac cycle using Echocardiographic methods. There are no published data on the cardiac phase-specific changes in the LA appendage volume indices. Therefore, we first examined the incremental LA and LAA volume changes per 10% increments of the cardiac cycle in a comparable age-matched cohort of 13 subjects (mean age of 79.7 ± 11 years). We used multiplane three-dimensional reconstructions of the multi detector CT images obtained at different phases of the cardiac cycle (0 to 80% R-R intervals) to accurately reflect the phase-specific volume change and a polynomial equation was derived from it to adjust the measured LA and LAA volume to 40% of the cardiac cycle (maximal volume of LA/LAA) which was used in the final analysis. The graphical illustration of LA and LAA volume changes per 10% of the cardiac cycle are shown in Figs. 2 and 3**,** respectively.

### Cardiac phase-specific volumetric measurements of the LA and LAA

**Fig. 1:**
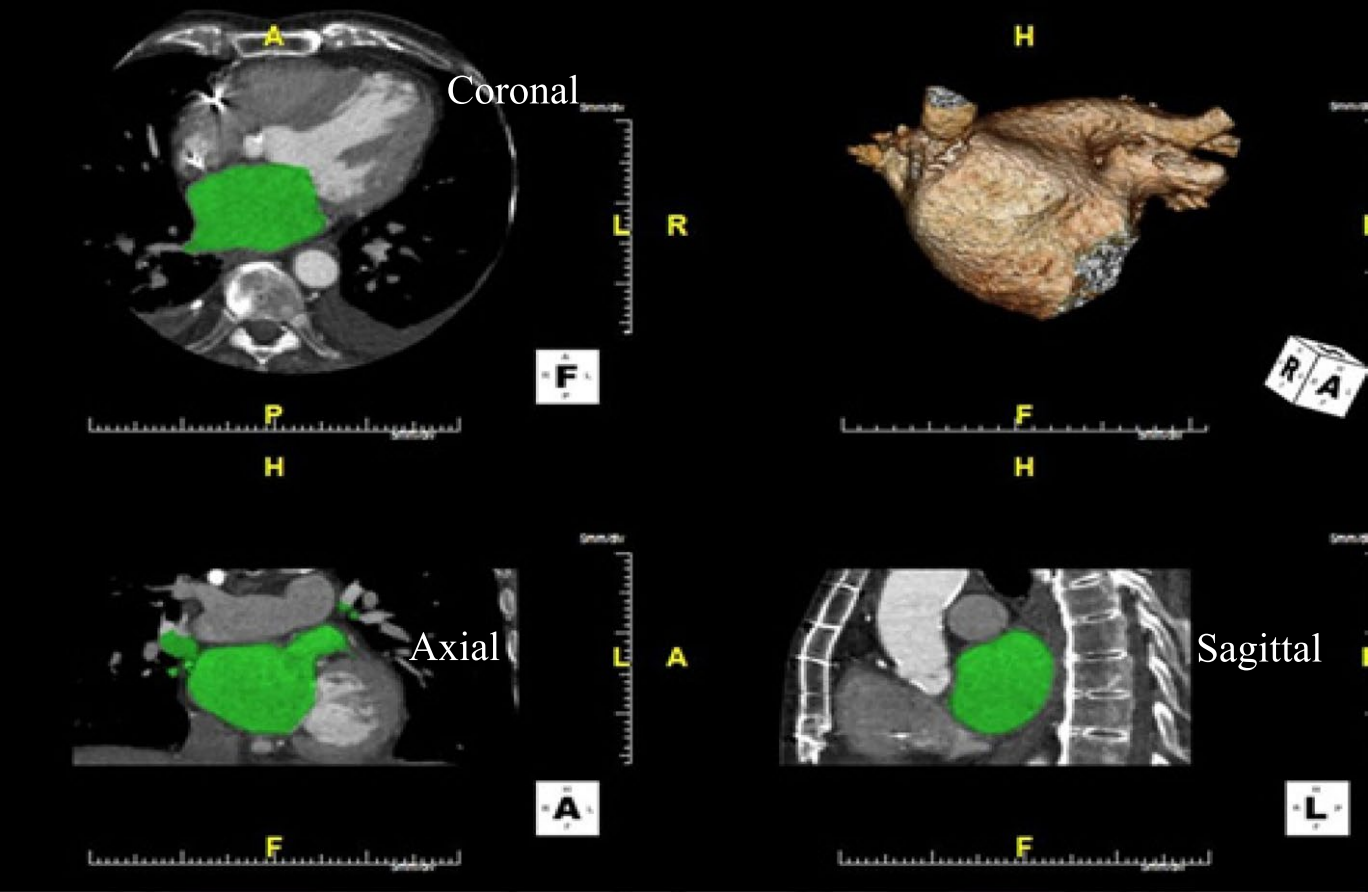
Volume acquisition and structural view of left atrium (LA) and left atrial appendage (LAA) obtained by using the three-dimensional multi-planar (axial, coronal and sagittal) reconstructions from multi-detector cardiac computer tomography. Determination of LA and LAA volume measures using three-dimension volume threshold-based method highlighted by the green zones. The top right images demonstrate the three-dimensional view of the LA, LAA and pulmonary veins

Volumetric measures of LA and LAA were obtained in between 30 and 80% of the R-to-R interval of ECG, where endocardial contours on the images could be sampled the best. These volumes obtained at specific phase of the cardiac cycle were adjusted to 40% of the R-R interval using the above derived eq. A second investigator independently calculated the volumetric measures and agreement were achieved in all cases. LA and LAA volumes were measured using the three-dimension volume-based threshold (3D VTB). For the 3D VTB method, the endocardial contours of the LA were semi-automatically traced on the axial slices using the analysis software. We applied the lowest value of CT attenuation to cover the entire contrast-enhanced LA cavity as well as to eliminate the pericardial fatty tissue within a region of interest. The lowest values of CT attenuation were determined when the LA cavity was completely included in the LA volume. The LAA volumes were calculated separately from the LA volume. We also calculated the pulmonary vein confluences from the volume calculations. Volume acquisition and structural view of LA and LAA obtained by using the three-dimensional multi-planar reconstructions from multi-detector cardiac CT is shown in Fig. 1.

### Clinical characteristics of the study subjects

Baseline patient characteristics including demographics, clinical symptomatology, surgical history, radiation history, laboratory data, medication use and Echocardiographic parameters were obtained from the chart review. Cardiac chamber measurements, left ventricular ejection fraction (LVEF), aortic valve area and left ventricular stroke volumes (LV-SVI) were obtained according to current American Society of Echocardiography recommended methods [18, 19]. Due the long natural history from the radiation exposure to aortic valve procedure, we retrieved only limited details of their cancer therapy protocols. Based on the available data, breast cancer (66%) was the commonest malignancy, followed by Hodgkin’s lymphoma (19%), lung cancer (7%), non-Hodgkin’s lymphoma (7%) and others (1%). Among breast cancer survivors 75% had left sided malignancy. There were no sufficient data available for the dose, frequency and stereotactic characteristics of the radiation exposure. The oncological characteristics of a similar larger cohort was previously published by our group [7].

### Follow up of clinical outcomes

In both radiation-exposed and control cohorts, the date of TAVR was considered as the beginning of the follow-up. All patients were routinely followed up after 30 days and 1 year at the structural heart clinic. The primary events were all-cause and CV mortality. We gathered the survival data from the medical record review, US social security Death Index or telephone follow-up. CV mortality was defined as any death associated to myocardial infarction, arrhythmia, heart failure, or sudden cardiac arrest. Secondary events were composite of MACE, defined as CV death, post-procedural acute myocardial infarction or revascularizations, heart failure exacerbation, and stroke until the date of last follow up.

### Statistical analysis

Categorical and continuous variables were reported as percentage and mean ± standard deviation (SD) respectively where appropriate. Baseline clinical and pre-procedural characteristics were compared between the two groups using the student’s t test or the Wilcoxon rank-sum test, as appropriate, for quantitative variables; and the Pearson chi-square test for categorical variables.

All relevant clinical, Echocardiographic, laboratory and pre-operative imaging variables were used in univariate cox proportional hazard analysis to determine the association with all-cause mortality. The variables that were significant (*p* < 0.05) on univariate analysis were used to construct the multivariate cox proportional hazard model. We used the false discovery rate (FDR) in multiple hypothesis testing to correct for multiple comparisons. In addition, the Kaplan-Meier survival curve for the LAA volume was constructed. A *p*-value of < 0.05 was considered statistically significant for all statistical analyses. All statistical analyses were performed using SAS statistical software, version 9.4 (SAS Institute Inc., Cary, NC).

## Results

### Comparison of baseline characteristics and echocardiographic data

Comparison of the baseline clinical and Echocardiographic data are shown in Table 1. There was no significant age difference between the radiation and control groups. Female gender was prevalent among the radiation group (CXRT group: 69.7%, non CXRT: 49.6%, *p* = 0.02), which likely reflects the higher proportion of breast cancer survivors in the CXRT group. Smoking, cerebrovascular accidents and use of aspirin were more common in the CXRT group. Both groups had identical surgical risk (STS score) scores at baseline. There was no significant difference in the left ventricular ejection fraction (LVEF) and mean aortic valve gradient between the two groups. Moderate to severe aortic, mitral and tricuspid regurgitation was seen in 32.8, 37.5 and 27.6% of the total study subjects, respectively. The incidence of moderate-severe mitral stenosis was disproportionately higher in the CXRT group.

**Table 1:** Baseline characteristics among CXRT and non-CXRT patients

Parameters	CXRT (***n***-43)	Non CXRT (***n***-141)	***P*** value
**Clinical variables**			
Age	80.9 ± 8.3	81.5 ± 9.2	0.72
BMI	26.9 ± 6.1	28.5 ± 6.2	0.14
Female, %	69.7	49.6	0.02*
White, %	95.4	93.6	0.39
STS score, mean	8.6 ± 4.5	8.3 ± 4.4	0.67
HTN, %	83.7	85.1	0.81
DM,%	34.8	29.7	0.57
HLD, %	69.7	56.0	0.12
Smoking, %	65.1	41.1	0.008*
CVA, %	55.8	4.3	< 0.0001*
COPD, %	34.9	43.3	0.38
CAD, %	62.8	51.1	0.22
ESRD, %	9.3	4.9	0.23
PAD, %	25.6	37.6	0.20
CABG, %	32.6	22.0	0.11
Cardiac surgery, %	32.7	26.2	0.44
3-coronary vessel disease, %	27.9	24.8	0.69
Ischemic cardiomyopathy (EF < 50%), %	14.0	19.1	0.50
Angina, %	34.9	28.4	0.45
Syncope, %	4.7	5.7	1.00
**Lab variables**			
Creatinine in mg/dl, mean	1.3 ± 1.0	1.3 ± 1.0	0.98
FEV1, mean	79.6 ± 24.7	74.1 ± 24.4	0.19
eGFR, mean	52.9 ± 22.5	53.9 ± 21.1	0.78
**Medications**			
Aspirin, %	81.6	61.7	0.02*
ACEI/ARB, %	21.1	35.5	0.11
Beta-blocker, %	75.6	78.7	0.67
Statins, %	69.8	56.0	0.11
**Echo Variables**			
LVEF, mean	57.6 ± 11.7	55.2 ± 13.4	0.29
Mean AV gradient	45.4 ± 14.7	43.8 ± 15.3	0.54
Mod-severe MS, %	13.9	3.6	0.02 *
Mod-severe MR, %	23.3	14.2	0.16
Mod-severe AR, %	18.6	14.2	0.47
Mod-severe TR, %	16.3	11.3	0.43

Values presented as *n* (%), mean (SD), or median (25-75th percentiles)

(*) indicates *p* valve < 0.05 for patients in the CXRT group compared to non CXRT group. *ACEI/ARB* angiotensin converting enzyme inhibitor/angiotensin receptor blocker, *AR* aortic regurgitation, *BMI* body mass index, *CAD* coronary artery disease, *CABG* coronary artery bypass graft, *COPD* chronic obstructive pulmonary disease, *CVA* cerebrovascular accident, *DM* diabetes mellitus, *ESRD* end stage renal disease, *eGFR* estimated glomerular filtration rate, *FEV1* force expiratory volume at 1 second, *HTN* hypertension, *HLD* hyperlipidemia, *PAD* peripheral arterial disease, *LVEF* left ventricular ejection fraction, *MS* mitral stenosis, *MR* mitral regurgitation, *STS* surgical thoracic society, *TR* tricuspid regurgitation

### Higher LA appendage volume index in patients with prior history of CXRT

The volumetric measures of the LA and LAA done at 40% of R-to-R interval of ECG gated cardiac CT are shown in Table 2. There were no significant differences in the LA and LAA volume between the CXRT and non-CXRT groups (*p* = 0.78 and 0.12, respectively). The LA volume index was also not different between the two comparison groups (*p* = 0.24). However, LAA volume index was found to be significantly higher in the CXRT group compared to non-CXRT group (CXRT:11.2 ± 8.9 ml/m^2^; non-CXRT:8.6 ± 4.5 ml/m^2^, *p* = 0.03). There were no significant differences in pulmonary venous ostial dimensions except for the right superior pulmonary vein, which was significantly higher in the non-CXRT group (*p* = 0.02).

**Table 2:** Volumetric measures of the LA and LAA at 40% of cardiac cycle

CCT variable	CXRT	Non-CXRT	***P*** value
LA volume, ml	140.9 ± 59. 5	138.3 ± 44.7	0.78
Indexed LA volume, ml/m^2^	83.6 ± 41.4	76.3 ± 26.8	0.24
LAA volume, ml	18.8 ± 13.8	15.7 ± 8.1	0.12
Indexed LAA volume, ml/m^2^	11.2 ± 8.9	8.6 ± 4.5	0.03*
RSPV ostium, cm^2^	2.7 ± 1.2	3.2 ± 1.1	0.02*
RIPV ostium, cm^2^	2.5 ± 0.9	2.8 ± 1.2	0.23
LSPV ostium, cm^2^	2.3 ± 0.9	2.6 ± 1.0	0.18
LIPV ostium, cm^2^	2.2 ± 1.0	2.2 ± 1.2	0.89

(*) *p*-value is significant, *LA* left atrial, *LAA* left atrial appendage, *LSPV* left superior pulmonary vein, *LIPV* left inferior pulmonary vein, *RSPI* right superior pulmonary vein, *RIPV* right inferior pulmonary vein

### Prolonged natural history and presence of multi-valvular lesions in the CXRT group

The median time from CXRT to TAVR was 19.0 years (Mean 20.1 ± 4.9 yrs.). Of the 43 symptomatic severe aortic stenosis patients with prior CXRT who underwent TAVR, breast cancer (66%) was the commonest reason for CXRT followed by Hodgkin’s lymphoma (19%), lung cancer (7%), non-Hodgkin’s lymphoma (7%) and others (1%). Among breast cancer survivors who had CXRT, 75% had left sided malignancy.

### Increased mortality and higher incidence of MACE in the CXRT group

Clinical outcomes stratified as the presence or absence of CXRT are shown in Table 3. During a median follow-up of 17.1 months, the all-cause mortality was found to be significantly higher in the CXRT group as compared to the control group (25.6% vs 9.2%, *p* = 0.009). The CV mortality was significantly higher in the radiation group compared to the non-radiation group (18.6% vs 2.84%, *p* = 0.001). Similarly, the incidence of MACE was significantly higher in the radiation group compared to the non-radiation group (55.81% vs 19.86%, p = < 0.0001).

**Table 3:** Post-TAVR outcomes stratified by the presence or absence of CXRT

Post-TAVR outcomes	CXRT	Non-CXRT	***P*** value
All-cause mortality, %	25.6	9.2	0.009*
MACE, %	55.81	19.86	< 0.0001*
CV mortality, %	18.6	2.84	0.001*

(*) *p*-value is significant. *MACE* major adverse cardiac events, *CV* cardiovascular

### Elevated LAA volume and LAA volume-index both predict mortality in the CXRT group

The results of univariate and multivariate cox proportional hazard analysis for all-cause mortality are shown in Table 4. The LAA volume at 40% of the cardiac cycle (HR: 1.03; 95% CI: 1.0 to 1.01, *p* = 0.01) and LAA volume index at 40% of the cardiac cycle (HR: 1.05; CI: 1.0 to 1.01, *p* = 0.03) were the strongest predictors of reduced survival in the CXRT group. Kaplan-Meier survival analysis showed reduced survival and increased incidence of MACE among those patients with elevated LAA volume of > 21.9 ml compared those with LAA volume of < 21.9 ml (adjusted to 40% of cardiac cycle) in Fig. 4**.**

**Table 4:** Fit Proportional Hazard Analysis for all-cause mortality

Univariate				Multivariate		
Parameters	RR	95% CI	P value	RR	95% CI	***P*** value
**Clinical variables**						
Age	0.97	0.93–1.02	0.35			
BMI	0.97	0.90–1.04	0.43			
White	0.70	0.09–5.27	0.73			
STS score, mean	1.03	0.94–1.11	0.43			
HTN, %	2.83	0.37–21.14	0.23			
DM,%	0.76	0.31–1.86	0.55			
HLD, %	1.14	0.50–2.62	0.74			
Smoking, %	1.72	0.74–3.97	0.19			
CVA, %	2.83	1.23–6.49	0.02*	1.85	0.90–3.82	0.09
COPD, %	1.35	0.60–3.03	0.47			
CAD, %	1.67	0.68–4.04	0.25			
ESRD, %	1.93	0.44–8.35	0.41			
PAD, %	1.69	0.74–3.86	0.21			
CABG, %	1.09	0.43–2.79	0.86			
Cardiac surgery, %	0.97	0.34–2.49	0.95			
3 Vessel Disease, %	0.96	0.38–2.44	0.94			
ICM (EF < 50%), %	2.23	0.89–5.54	0.10			
Angina, %	0.88	0.36–2.14	0.78			
Syncope, %	2.28	0.65–7.95	0.24			
**Lab Variables**						
Creatinine in mg/dl, mean	1.2	0.91–1.54	0.14			
FEV1, mean	0.98	0.96–1.01	0.07			
eGFR, mean	0.99	0.97–1.01	0.66			
**Echo and CCT variables**						
Mod -severe MS, %	2.74	0.91–8.24	0.11			
Mod-severe AR, %	1.02	0.36–2.85	0.97			
Mod-severe MR, %	1.16	0.43–3.14	0.76			
Mod-severe TR, %	1.60	0.60–4.27	0.36			
LAV, ml	1.00	0.99–1.02	0.13			
LAVI, ml/m^2^	1.00	0.99–1.02	0.36			
^#^LAAV, ml	1.05	1.01–1.08	0.009*	1.03	1.0–1.01	0.01*
^#^LAAVI, ml/m^2^	1.07	1.0–1.13	0.03*	1.05	1.0–1.01	0.03*
RSPV ostium, cm^2^	0.85	0.57–1.21	0.39			
RIPV ostium, cm^2^	1.31	0.93–1.78	0.12			
LSPV ostium, cm^2^	0.72	0.45–1.08	0.12			
LIPV ostium, cm^2^	1.12	0.79–1.41	0.45			
LVEF	0.98	0.95–1.02	0.42			
Mean AV gradient, mmHg	1.01	0.98–1.04	0.49			
**Medications**						
Aspirin, %	1.20	0.49–2.91	0.67			
ACEI/ARBs, %	0.78	0.31–1.98	0.61			
Beta blocker, %	0.82	0.30–2.21	0.69			
Statins, %	1.14	0.50–2.62	0.74			

#, Only one variable modeled during multivariate analysis as both represented the similar data

Values presented as *n* (%), mean (SD), or median (25-75th percentiles)

(*) indicates *p* valve < 0.05 for patients in the CXRT group compared to non CXRT group. *ACEI/ARB* angiotensin converting enzyme inhibitor/angiotensin receptor blocker, *AR* aortic regurgitation, BMI body mass index, *CAD* coronary artery disease, *CABG* coronary artery bypass graft, *COPD* chronic obstructive pulmonary disease, *CVA* cerebrovascular accident, *DM* diabetes mellitus, *ESRD* end stage renal disease, *eGFR* estimated glomerular filtration rate, *FEV1* force expiratory volume at 1 second, *HTN* hypertension, *HLD* hyperlipidemia, *ICM* ischemic cardiomyopathy, *LAV* left atrial volume, *LAVI* left atrial volume index, *LAAV* left atrial appendage volume, *LAAVI* left atrial appendage volume index, *PAD* peripheral arterial disease, *LVEF* left ventricular ejection fraction, *MS* mitral stenosis, *MR* mitral regurgitation, *STS* surgical thoracic society, *TR* tricuspid regurgitation

## Discussion

This is the first observational study that is uniquely designed to utilize standard cardiac-gated CT-based 3-D volume measurement protocols for the accurate volumetric quantification of LA, LAA, pulmonary venous ostia, and consequently use these parameters as the risk predictors of mortality in cancer survivors with a prior exposure to thoracic radiation. This is also the first study to develop a cardiac-phase-specific volume quantification algorithm to accurately calculate the LA and LAA volume indices using 3-D volume rendered cardiac CT.

The prognostic data derived from newly developed LA and LAA volume-quantification algorithms are clinically important. Our data expand on the prior studies that reported that LAA can retain larger blood volume when the LA pressure is elevated [12, 13]. In particular, we report that prior chest radiation leads to significantly elevated LAA volume-index compared to the control population. This can either reflect chronically elevated LA pressure, or a direct effect in the LAA since the LAA is more anteriorly located structure, and is exposed to higher radiation dose when common cancers including breast cancers are irradiated. However, since there is paucity of data comparing the volumetric changes of the LA vs.*,* LAA in fibrotic myocardial remodeling as expected with ionizing radiation, larger prospective longitudinal studies are needed to determine whether LAA volume index is indeed a better risk predictive marker for mortality. In our study with a modest sample size, both LA and 237 LAA volumes showed a weak but positive correlation with the body surface area (Fig. 5). The implications of these findings are that, if validated in larger cohorts, LAA volumetric indices normalized to body surface area can be better predictors of clinical outcomes.

Our finding of a higher all-cause and CV mortality in radiation group confirms the prior reported findings of cardiac effects from radiation including cardiomyocytes injury, myocardial fibrogenesis, coronary microvasculature damage and valvular heart diseases [20–22]. Changes in LA and LAA size can be typically attributed to mitral stenosis. In our previously published study, when mitral stenosis was studied as a variable affecting LA/LAA size, significant differences were present in the univariate analysis. However, multivariate analysis showed no statistical significance (*p* > 0.05) [7]. Overall, these pathologies contribute chronic myocardial dysfunction leading elevated LV end-diastolic pressure, which in turn can elevate LA pressure [23, 24]. Since, LAA and pulmonary ostia are in direct continuity to the LA, the elevated LA pressure can also promote LAA and pulmonary venous ostial remodeling. This also explains an increased incidence atrial arrhythmia including atrial fibrillation in patients with prior cardiac irradiation [7, 25]. Unfortunately, we were not able to include patients with ongoing atrial fibrillation, since cardiac CT imaging in these patients was less than optimal due to ECG gating-artifacts for accurate volumetric assessments at different phases of the cardiac cycle.

**Fig. 2:**
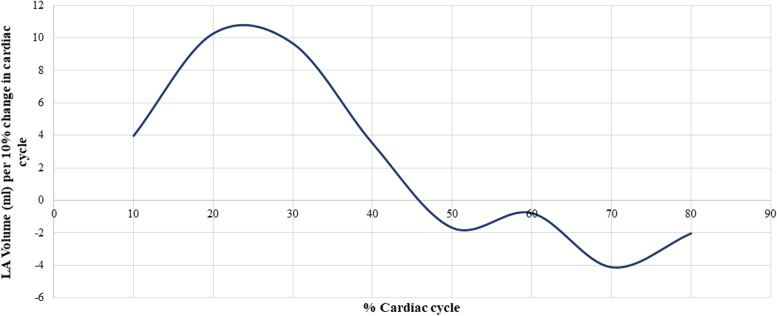
Left atrial (LA) volume curve. The x-axis represents the percent change in the cardiac cycle, and y-axis represents the volume change in the LA. The acquisition of LA volume was performed using three-dimensional reconstructions of the multi detector electrocardiographic gated cardiac computed tomography (CT) images obtained at different phases of the cardiac cycle (0 to 80% R-R interval) to reflect incremental volume change per 10% change in cardiac cycle

**Fig. 3:**
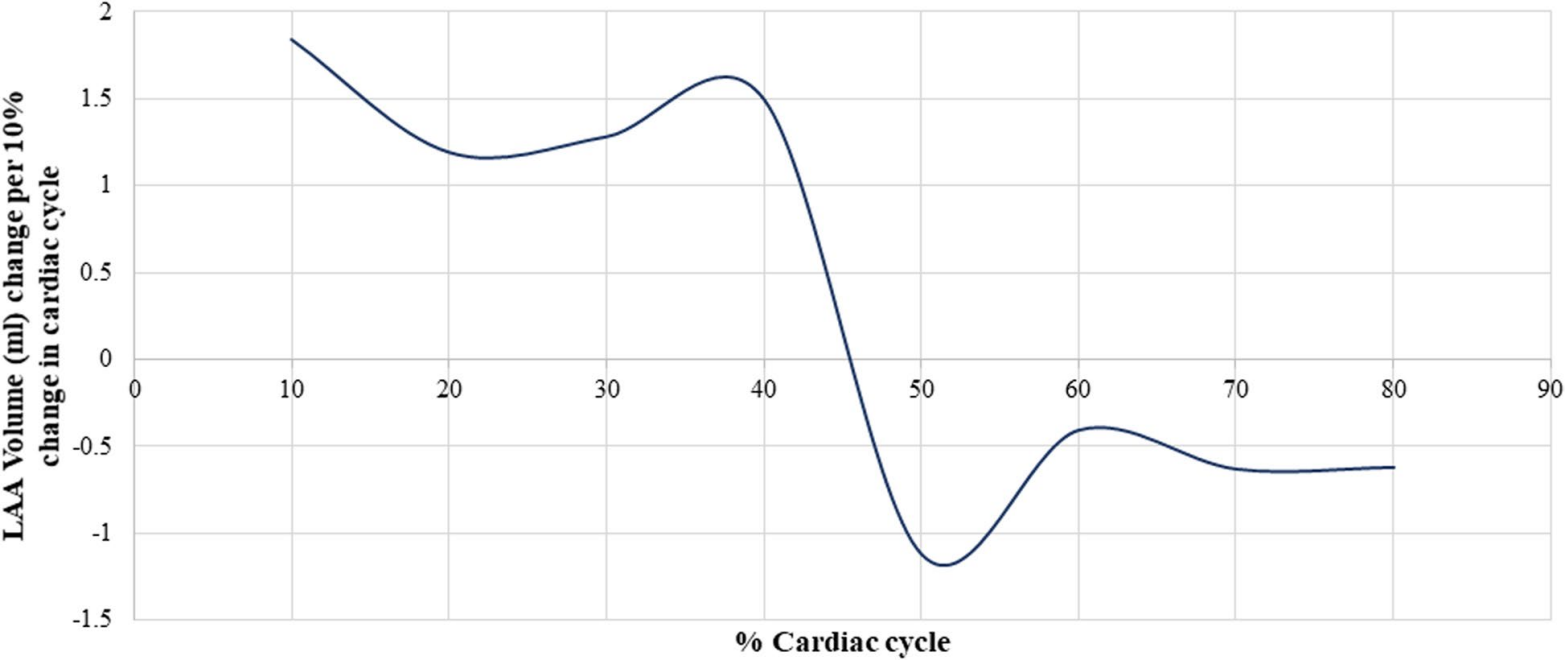
Left atrial appendage (LAA) volume curve. The x-axis represents the percent change in the cardiac cycle, and y-axis represents the volume change in the LAA. The acquisition of LAA volume was performed using three-dimensional reconstructions of the multi detector electrocardiographic gated cardiac computed tomography (CT) images obtained at different phases of the cardiac cycle (0 to 80% R-R interval) to reflect incremental volume change per 10% change in cardiac cycle

Our Kaplan-Meier survival analysis showed reduced survival and increased incidence of adverse cardiac events among those patients with elevated LAA volume of > 21.9 ml compared those with LAA volume of < 21.9 ml (adjusted to 40% of the cardiac cycle). Since there are no published data on the cardiac phase-specific changes in the LA appendage volume indices, we first examined the incremental LA and LAA volume changes per 10% increments of the cardiac cycle in a comparable age-matched cohort of 13 subjects (mean age of 79.7 ± 11 years) (Figs. 2 and 3). Our results have shown that the patients that underwent radiation exposure had significantly higher LA size, compared to the controls. However, these data should be interpreted with a caution since, compared to age-matched controls, cancer patients are exposed to various other therapies including anthracyclines. Additionally, radiation can affect other heart valves including the mitral valve, which can also lead to elevated LA size.

**Fig. 4:**
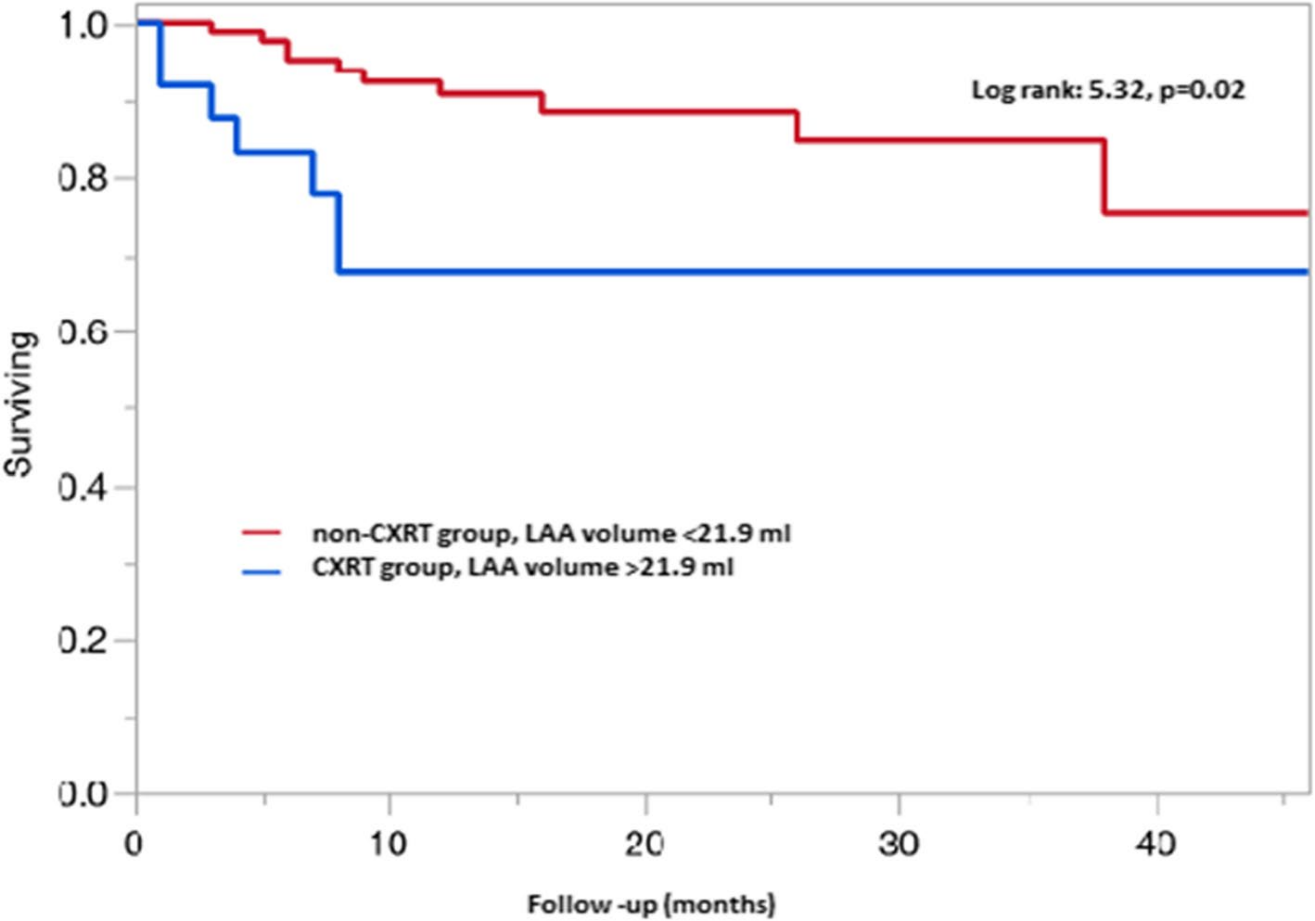
Kaplan Meir survival curve for the mortality rate in patients with and without prior chest radiation therapy. Reduced survival in CXRT group with elevated LAA volume of more than 21.9 ml compared to those with LAA volume of less than 21.9 ml adjusted to 40% of the cardiac cycle. The red line denotes patients without prior chest radiation therapy (non-CXRT group) and blue line denotes patients with prior chest radiation therapy (CXRT group)

**Fig. 5:**
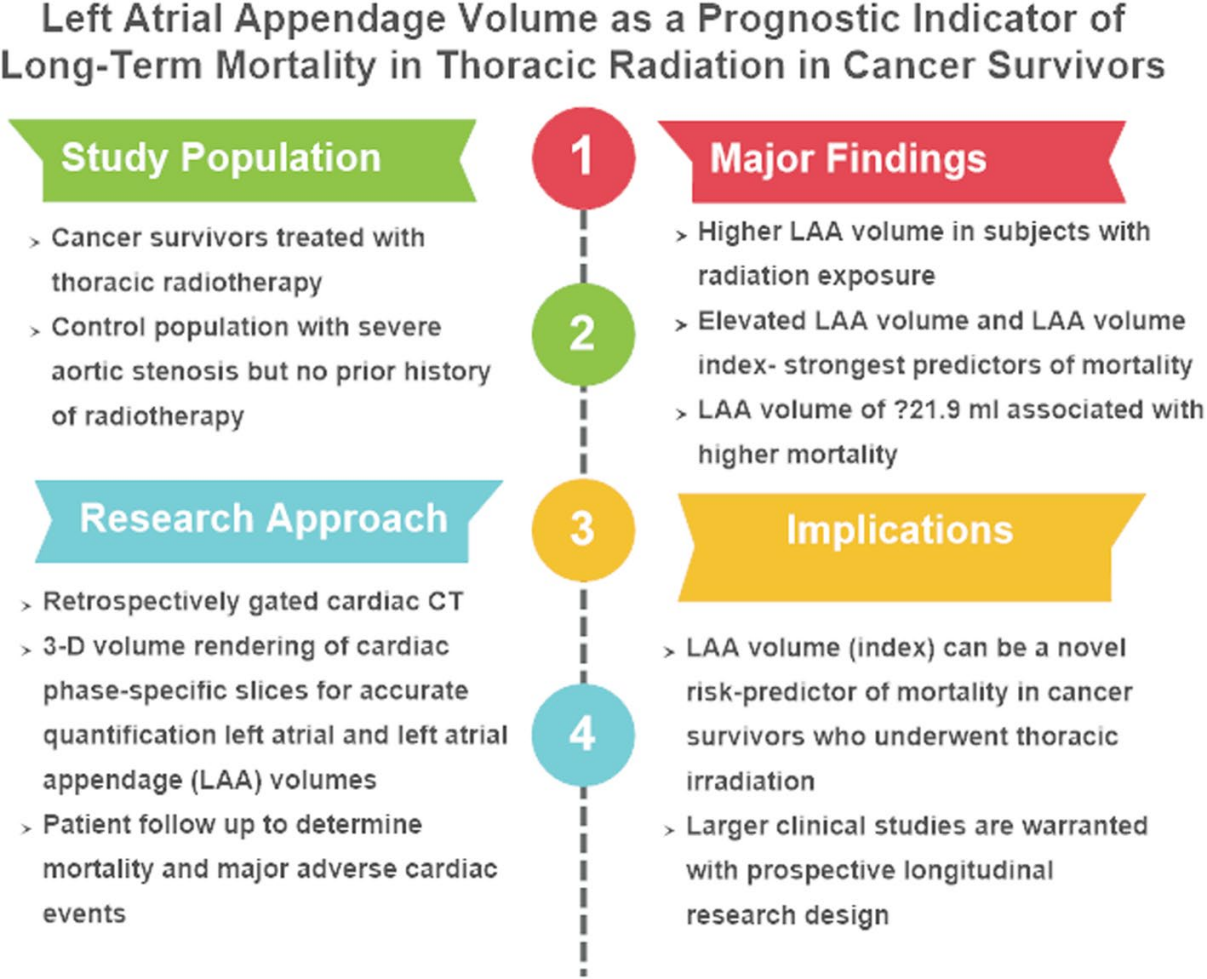
Graphical Abstract summarizing the study design, approach, major findings, and their clinical implications

To sum up, our study addresses an important question of immediate clinical significance in cancer survivors treated with chest radiation. We believe that this is the first observational study to analyze the prognostic value of cardiac CT derived atrial volumes in cancer survivors presenting with severe valvular heart disease. To accomplish this goal, we have also developed a CT-based algorithm to calculate LA and LAA volumes at different phases of cardiac cycle. Such algorithms enhance accuracy and facilitate better comparison of atrial volumes during the atrial filling phase of the cardiac cycles. Compared to transthoracic echocardiogram, cardiac CT estimates LA or LA appendage volumes with no geometric assumptions and thereby provides more accurate evaluations [26]. An overview of research aims, study findings and the clinical implications have been outlined in Fig. 5.

## Limitations

We acknowledge the following limitations. First, both radiotherapy and chemotherapy are frequently used in the multidisciplinary management of cancer patients and it is expected that our study patients were also exposed to such combined treatment modalities. Given ~ 20-year natural history for the development of valvular disease after radiation exposure, we were not able to retrieve the complete chemotherapy records from most of our study subjects. Therefore, this study could not clearly discern the role of systemic chemotherapies (including anthracyclines, fluorouracil and trastuzumab) contributing to the development of cardiac dysfunction and LA/LAA remodeling. Other confounding factors in this study include lack of detailed information on the tumor types, radiation doses, radiotherapy frequency, and stereotaxic data from all patients.

Second, we excluded subjects with atrial fibrillation since their CT images showed significant gating artifacts. Third, our study population shows a demographic heterogeneity (females>males) and we were not able to specifically examine radiation frequency and dosage in various subsets of patients with thoracic malignancies. This would obligate access to a larger patient database for future prospective studies.

## Conclusion

The cardiac CT derived LAA volume indices are robust markers of mortality and cardiovascular events in cancer survivors with prior CXRT. Our findings suggest that LA and LA appendage volume assessment should be part of the routine cardiac evaluation whenever patients undergo CT-based cardiovascular evaluation. The implications these data should be taken into consideration when discussing the clinical management plan for these patients.

## Data Availability

The original contributions presented in the study are included in the article/supplementary material, further inquiries can be directed to the corresponding author.

## Abbreviations

LA: Left atrium
LAA: Left atrial appendage
CXRT: Chest radiotherapy
MACE: Major adverse cardiac events
